# Detection of heart rate using smartphone gyroscope data: a scoping review

**DOI:** 10.3389/fcvm.2023.1329290

**Published:** 2023-12-18

**Authors:** Wenshan Wu, Mohamed Elgendi, Richard Ribon Fletcher, Hagen Bomberg, Urs Eichenberger, Cuntai Guan, Carlo Menon

**Affiliations:** ^1^ Biomedical and Mobile Health Technology Lab, Department of Health Sciences and Technology, ETH Zurich, Zurich, Switzerland; ^2^ School of Computer Science and Engineering, Nanyang Technological University, Singapore, Singapore; ^3^ Department of Mechanical Engineering, Massachusetts Institute of Technology, Cambridge, MA, United States; ^4^ Department for Anesthesiology, Intensive Care and Pain Medicine, Balgrist University Hospital, Zürich, Switzerland

**Keywords:** heart rate monitor, smartphone application, mobile health, digital health, seismocardiography, GCG technology, smart healthcare, remote monitoring

## Abstract

Heart rate (HR) is closely related to heart rhythm patterns, and its irregularity can imply serious health problems. Therefore, HR is used in the diagnosis of many health conditions. Traditionally, HR has been measured through an electrocardiograph (ECG), which is subject to several practical limitations when applied in everyday settings. In recent years, the emergence of smartphones and microelectromechanical systems has allowed innovative solutions for conveniently measuring HR, such as smartphone ECG, smartphone photoplethysmography (PPG), and seismocardiography (SCG). However, these measurements generally rely on external sensor hardware or are highly susceptible to inaccuracies due to the presence of significant levels of motion artifact. Data from gyrocardiography (GCG), however, while largely overlooked for this application, has the potential to overcome the limitations of other forms of measurements. For this scoping review, we performed a literature search on HR measurement using smartphone gyroscope data. In this review, from among the 114 articles that we identified, we include seven relevant articles from the last decade (December 2012 to January 2023) for further analysis of their respective methods for data collection, signal pre-processing, and HR estimation. The seven selected articles’ sample sizes varied from 11 to 435 participants. Two articles used a sample size of less than 40, and three articles used a sample size of 300 or more. We provide elaborations about the algorithms used in the studies and discuss the advantages and disadvantages of these methods. Across the articles, we noticed an inconsistency in the algorithms used and a lack of established standardization for performance evaluation for HR estimation using smartphone GCG data. Among the seven articles included, five did not perform any performance evaluation, while the other two used different reference signals (HR and PPG respectively) and metrics for accuracy evaluation. We conclude the review with a discussion of challenges and future directions for the application of GCG technology.

## Introduction

Heart rate (HR) measurement is essential for detecting pathological irregularities, indicative of heart-related conditions such as arrhythmia (i.e., atrial fibrillation (AFib)), myocardial ischemia, and heart failure (1–7). These conditions often manifest through pathological irregularities in HR, underscoring the importance of HR monitoring in cardiovascular health. Cardiovascular diseases account for approximately 32% of all global deaths annually (8), necessitating effective monitoring and early intervention. Regular HR monitoring is pivotal for the timely treatment and prevention of various cardiovascular risk factors (9).

Beyond cardiovascular health, HR measurement also plays a role in assessing mental activities and stress disorders, useful in gauging mental workload (10) and diagnosing stress-related conditions (11). HR monitoring is increasingly utilized for fitness tracking and exercise intensity monitoring, making it an integral part of both physical and mental health assessments.

Traditionally, HR measurement often involves electrocardiography (ECG), a direct method for HR detection. However, the interpretation of ECG data requires specific expertise, distinguishing it from other sensors like MEMS gyro (12). ECG measurements are not ideal for long-term daily HR monitoring due to practical challenges like irritation from electrode gels and signal quality issues with dry/capacitive electrodes. This has led to the emergence of various commercial non-invasive HR monitors (13).

The widespread prevalence of smartphones (14) has spurred innovations in smartphone-based heart rate (HR) estimation systems (15, 16). Smartphone-based ECG systems often involve electrodes, which have been noted for causing skin irritation in some cases (17). In contrast, smartphone-based photoplethysmography (PPG) operates in a non-invasive manner without the risk of skin irritation (18). Commonly used in wearable devices, PPG measures heart rate by detecting changes in blood volume within peripheral arterioles during each cardiac cycle. It utilizes a light source and a photodetector placed on the skin to capture variations in light absorption due to pulsatile blood flow, thereby facilitating accurate heart rate measurement (19–21).

PPG, however, has its limitations in HR measurement accuracy due to noisy signals and susceptibility to motion artifacts and environmental light variations. Furthermore, PPG provides limited insight into the mechanoelectrical feedback and biomechanics of cardiac electromechanical coupling (22, 23).

Advancements in microelectromechanical systems (MEMS) (24), now common in modern smartphones, have led to the utilization of MEMS sensors in smartphone-based HR monitoring systems (25, 26). Mechanocardiography (MCG), which includes gyrocardiography (GCG), seismocardiography (SCG), and ballistocardiography (BCG), measures cardiac mechanics induced by heartbeats (27). GCG measures vibrations of the chest wall due to cardiac activity using gyroscopes (28), while SCG and BCG use accelerometers (ACCs) to measure cardiac vibrations (29) and the recoil forces of ejected blood (30), respectively. The increase in MCG studies in recent years (31–34) highlights its potential in cardiac performance assessment.

However, most smartphone-based HR monitoring algorithms overlook GCG signals. Despite the significant contribution of gyration signals to cardiac vibrational energy (35) and their independence from gravity’s effects (36), few studies have explored GCG for HR estimation. This lack of standardization in reference signals and metrics for GCG performance evaluation presents a gap in the literature.

This work presents a scoping review of publications concerning HR estimation using smartphone GCG data, aiming to assess the current literature and guide future research in this field.

## Methods

### Search terms

A literature search was done using six different search engines: Springer, Embase (Elsevier), Cochrane Library (Wiley), EBSCOhost, IEEE Xplore, and PubMed. The search followed the PRISMA guidelines for scoping reviews (37). In addition to using the search terms combined with logical operators such as “OR” for union, “AND” (“&”) for intersection, and “−” for negation, we also searched the reference lists of the relevant articles to perform a more thorough literature search. To capture the latest developments in this area, we limited our search to results published between December 2012 and January 2023. The search terms used and search results are summarized in Table 1 below.

**Table 1:** Summary of search terms used.

Search engine	Search term	Constraints	# Results
Springer	(((vibrational cardiography) OR (gyroscope) OR (gyrocardiography) OR (gcg) OR (mechanocardiogram)) AND ((heartrate) OR (heart rate) OR (cardio) OR (cardiography)) AND ((smartphone) OR (phone) OR (mobile)))	Title must contain ’phone’, 2012–2023	4
Embase (Elsevier)	(((vibrational cardiography) OR (gyroscope) OR (gyrocardiography) OR (gcg) OR (mechanocardiogram)) AND ((heartrate) OR (heart rate) OR (cardio) OR (cardiography)) AND ((smartphone) OR (phone) OR (mobile)))	2012–2023	28
Cochrane Library (Wiley)	(((vibrational cardiography) OR (gyroscope) OR (gyrocardiography) OR (gcg) OR (mechanocardiogram)) AND ((heartrate) OR (heart rate) OR (cardio) OR (cardiography)) AND ((smartphone) OR (phone) OR (mobile)))	Search terms applied to title, abstract, and keyword, 2012–2023	5
EBSCOhost	((vibrational cardiography) OR (gyroscope) OR (gyrocardiography) OR (gcg) OR (mechanocardiogram)) Abstract AND ((heartrate) OR (heart rate) OR (cardio) OR (cardiography)) Title AND ((smartphone) OR (phone) OR (mobile))	Search terms applied to title, 31 Dec 2012 to 01 Jan 2023	1
IEEE Xplore Digital Library database	(“Abstract”:vibrational cardiography OR “Abstract”:gyroscope OR “Abstract”:gyrocardiography OR “Abstract”:gcg OR “Abstract”:mechanocardiogram) AND (“Document Title”:heartrate OR “Document Title”:heart rate OR “Document Title”:cardio OR “Document Title”:cardiography) AND (“Document Title”:smartphone OR “Document Title”:phone OR “Document Title”:mobile)	2012–2023	4
PubMed	(((vibrational cardiography) OR (gyroscope) OR (gyrocardiography) OR (gcg) OR (mechanocardiogram)) & ((heartrate) OR (heart rate) OR (cardio) OR (cardiography)) & ((smartphone) OR (phone) OR (mobile)))	2012–2023	20
Searching the reference lists	N.A.	2012–2023	52

# Results, number of results; N.A., not applicable.

The employment of six different databases was necessitated by the diverse range of publications and the varying scope of each database. Due to the unique search functionalities and limitations inherent to each database, distinct constraints were judiciously applied to each to maximize the relevance and quality of the search results. This approach was designed to ensure a thorough and exhaustive coverage of the literature in the rapidly evolving field of gyrocardiography. Furthermore, the search of the reference lists was conducted in an iterative manner. Initially, we performed a primary review of the references from the first round of results. Subsequent rounds of review were then conducted, with each round delving deeper into the references of the previously identified studies. This iterative process allowed us to capture a broader spectrum of relevant studies, thereby enriching the comprehensiveness of our review and ensuring that key contributions in the field were not overlooked. By adopting this meticulous and multi-faceted search strategy, we aimed to address the complexities of research dissemination in this specialized area of study, thereby providing a robust and comprehensive overview of the current state of gyrocardiography research.

## Results

**Figure 1:**
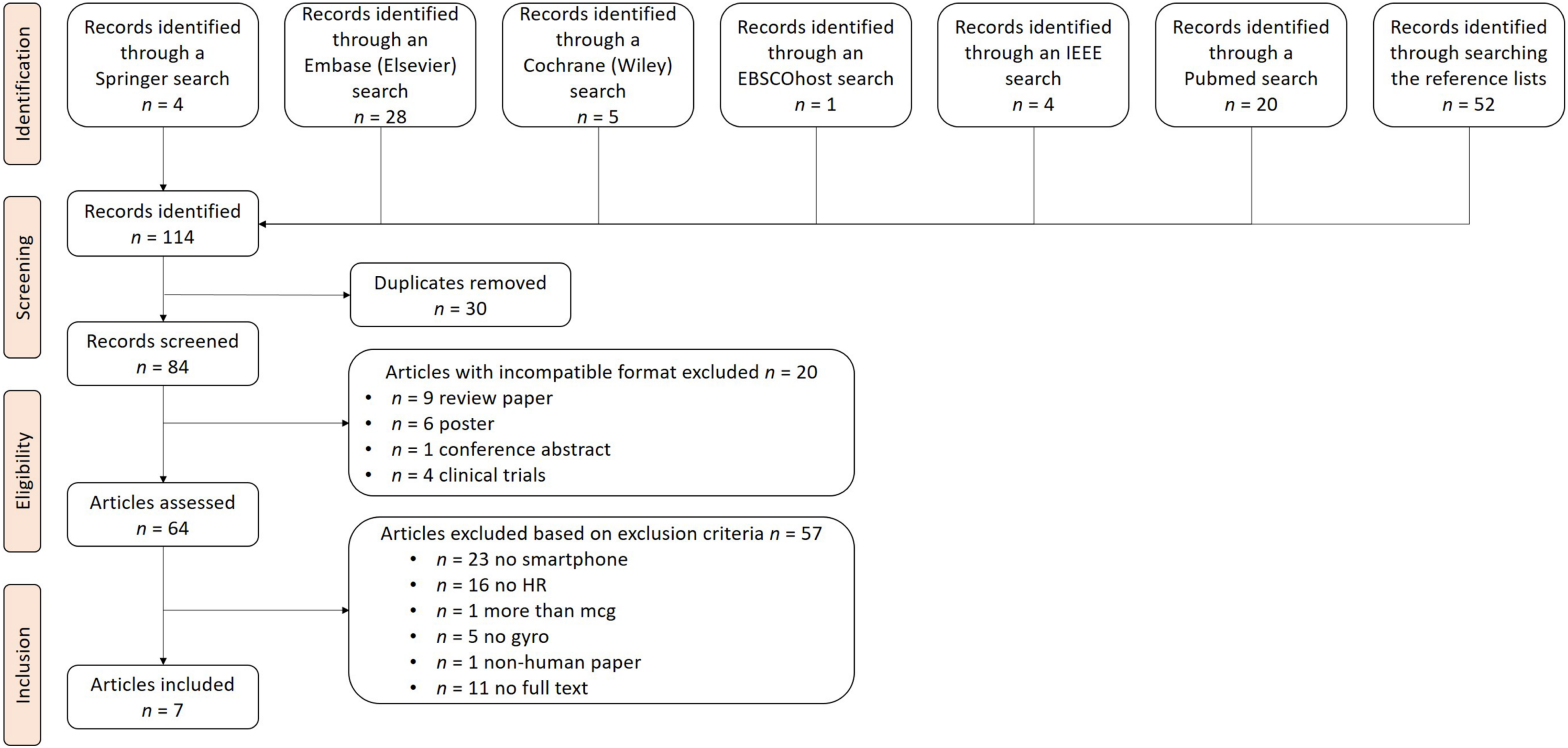
PRISMA flow chart. This figure illustrates the search process, which consisted of the identification, screening, eligibility checking, and inclusion of articles, where n stands for the number of articles at each step. After our search, seven articles were included for further analysis.

We identified 7 potential studies out of 114 studies retrieved and assessed in full-text according to our inclusion criteria as shown in Figure 1. During the screening process, 30 duplicates and 20 articles with incompatible formats were excluded. The remaining 64 articles were assessed for eligibility. A set of exclusion criteria was used to select the articles to be included in the review. Among the 64 articles, 57 were considered irrelevant due to the following reasons: 23 did not use smartphone sensors in their data collection process, 16 did not derive HR using the MCG signals, five did not use GCG, one used additional signals along with MCG, and one conducted experiments concerning the HR of non-human subjects (pet dogs). Finally, a further 11 articles without full-text access were excluded. As a result of the filtering process, we included seven articles in our detailed analysis.

An overview of the selected publications is provided in the publication’s subsection below. The subsection on demographic data describes the subjects involved in the data acquisition process of the experiments. The methodology subsection scrutinizes the different methodologies used, as well as the evaluation metrics used for HR extraction. Finally, the discussion summarizes the results, describes challenges, and proposes directions for future research.

### Publications

Seven publications were included for further analysis. Table 2 summarizes the essential aspects of the included publications. Only two (38, 39) of these publications focused precisely on HR estimation using MCG data and conducted performance evaluations for the estimations, while the other five (22, 40–43) derived HR from MCG data but used HR as a feature for downstream classification of heart conditions such as AFib. Therefore, these five articles did not provide any evaluation metrics for HR estimation. Nevertheless, these articles were included because they describe in detail the processing and methodology required to obtain HR from MCG data. Six of the included articles (22, 38, 40–43) were authored by a consistent group of individuals. Consequently, an inquiry into the dissimilarities pertaining to the datasets and methodologies employed in the articles to estimate HR was conducted and the results are presented in the dataset demographics section below (for the datasets) and the discussion section (for the methodologies).

**Table 2:** Summary of content of all publications included in the review.

Publication			Data Acquisition				Algorithm		Evaluation
Author (year)	# subjects (F:M)	# recordings	Smartphone model	Built-in sensors (Fs)	Subject position & sensor site	Recordings details	Summarized methodology	Reference (device)	HR Error${}^{a}$
Lahdenoja et al. (40)	39 (5:34)	39	Sony Xperia Z-series	ACC & GYRO (200 Hz)	Supine position with the smartphone placed on the chest; the phone’s loudspeaker points toward the subject’s head, and its display faces up so that the ACC-Z axis is parallel to the force of gravity	Each recording comprises 6-axis MCG data of approximately 2 minutes	Remove artifacts from the bandpass-filtered FFT values; peak detection on cross-correlation results of overlapping signal segments to obtain heartbeat intervals	N/R	N/R
Jaakkola et al. (41)	300 (132:168)	300	Sony Xperia	ACC & GYRO (200 Hz)	Smartphone is placed on the sternum of the subject	Each recording comprises 3-minute 6-axis MCG and synchronized 5-lead telemetry ECG signals	N/R	ECG (Philips IntelliVue MX40${}^{d}$)	N/R
Iftikhar et al. (42)	105 (29:70)${}^{b}$	105	Sony Xperia Z series	ACC & GYRO (200 Hz)	Supine position with the smartphone placed on the sternum bone	Each recording comprises 6-axis MCG data of up to 3 minutes	Breathing component removal from the bandpass-filtered FFT values; peak detection on cross-correlation results of overlapping signal segments to obtain heartbeat intervals	ECG	N/R
Tadi et al. (22)	435 (150:190)${}^{b}$	435	Sony Xperia Z1 and Z5	ACC & GYRO (200 Hz)	Supine position with the smartphone placed longitudinally on the chest; the phone’s screen faces upwards and its bottom edge is at the level of the lower edge of the sternum; this captures lateral movements (x-axis), head-to-foot aligned movements (y-axis), and dorsoventral movements from precordial vibrations (z-axis)	Each recording comprises 3-minute 6-axis MCG and synchronized 5-lead telemetry ECG signals	Noise removal by SSA; use of triangular moving-average filters to detect envelope; FFT and short-term autocorrelation used on the segmented signals before finding the first side peak	ECG (Philips IntelliVue MX40${}^{d}$)	N/R
Mehrang et al. (43)	300 (132:168)${}^{c}$	300	Sony Xperia Z1 and Z5	ACC & GYRO (200 Hz)	Supine position with the smartphone placed longitudinally on the bare chest; the phone’s screen faces upwards and its bottom edge is at the level of the lower edge of the sternum	Each recording comprises a 3-minute 6-axis MCG and synchronized 5-lead telemetry ECG signals	Breathing component removal from the bandpass-filtered FFT values; peak detection on cross-correlation results of overlapping signal segments used to obtain heartbeat intervals	ECG (Philips IntelliVue MX40${}^{d}$)	N/R
Hurnanen et al. (38)	66 (15:51)	66	N/R	ACC & GYRO (200 Hz)	Supine position with the smartphone placed on the upper chest and the PPG sensor placed on the left index finger	Each recording comprises a 2-minute 6-axis MCG signals, with synchronized PPG signals as reference	Triangular filter used for bandpass-filtered signals; the dynamic range of signals balanced using SMQT algorithm; negative values of the median-filtered values truncated; peak detection to estimate HR	PPG (Nordic 32-bit ARM Cortex-M0 CPU & RFD22301 Bluetooth)	0.87% missed, 0.31% false, 1.18% total
Mohamed et al. (39)	11 (6:5)	836	Samsung Note II, Samsung S5 and Sony Z2	GYRO (100 Hz)	Subject holds the smartphone over the chest by hand and can be sitting (default) or lying down${}^{e}$	N/R	Local mean removal to reduce noise; bandpass filter FFT values to obtain instantaneous HR; merge axes by a Kalman filter weighted by a quality metric; obtain HR estimation with an α-trimmed mean filter	HR (Omron HEM-432C)	1.03 bpm MedAE

#, number of; F:M, female : male; Fs, sampling frequency; N/R, not reported; SSA, single spectrum analysis; FFT, fast Fourier transform; ECG, electrocardiogram; ACC, accelerometer; GYRO, gyroscope; PPG, photoplethysmogram; MCG, mechanocardiogram; CPU, microprocessor; SMQT, successive mean quantization transform; HR, heart rate; bpm, beats per minute, MedAE, median absolute error.

${}^{a}$Error rate of HR detection only.

${}^{b}$Some subjects were unregistered.

${}^{c}$Inconsistent reporting of gender count.

${}^{d}$Reference signals were used for visualization only, not for quantitative evaluation.

${}^{e}$The smartphone can be held in either horizontal (default) or vertical orientation with respect to the ground.

### Demographics of the datasets

**Figure 2:**
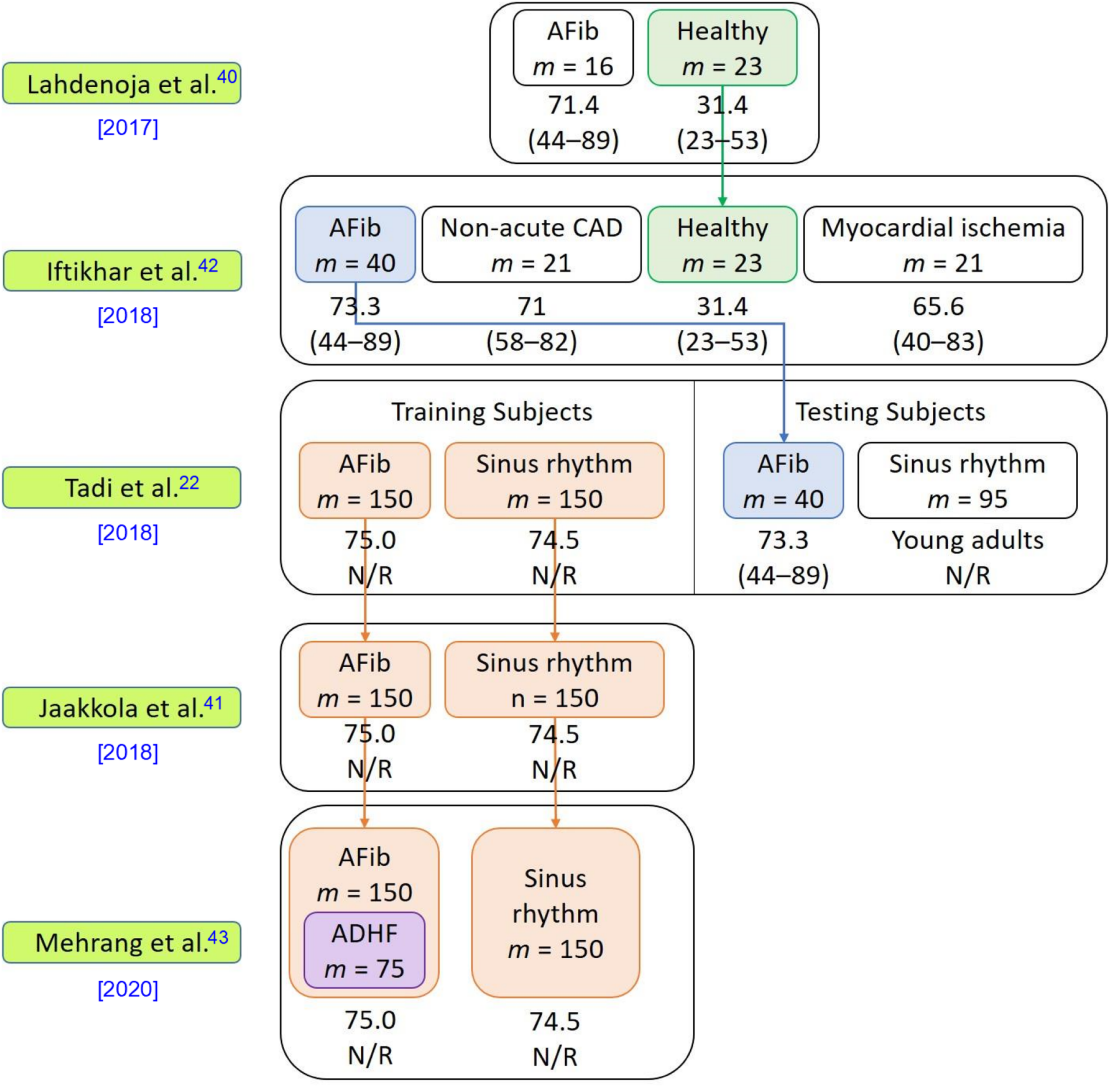
Dataset information used in the five included studies. This figure presents details of the datasets used in the articles, including the number of subjects, subjects’ comorbidities, and the relationships among the datasets in each publication. The symbol “m” denotes the number of subjects in each respective category. The average age of each cohort is presented beneath the cohort name, with the age range given in parentheses “()” below the average age. Abbreviations used in the figure include atrial fibrillation (AFib), coronary artery disease (CAD), acute decompensated heart failure (ADHF), and not reported (N/R). The downward arrow ↓ signifies the reuse of a dataset in subsequent studies.

This section analyzes the articles in terms of their demographics, including sample size, age distribution, gender distribution, and comorbidities. Some of the articles (22, 40–43) reused previous contributions to the datasets as shown in Figure 2.

The selected articles’ sample sizes varied between 11 and 435 participants, with two studies (39, 40) having a sample size of fewer than 40 participants and three studies (22, 41, 43) having a sample size of 300 participants or more. All articles reported the gender distribution of the subjects. Two articles (38, 40) used samples with skewed gender ratios where more than 80% of the subjects were male. Among the included articles, four (22, 40, 42, 43) provided the body mass index (BMI) statistics of the subjects, which may offer insights into the relationship between obesity and cardiovascular health conditions (44). However, BMI alone is regarded as a weak indicator of the percent of body fat and its negative influence on health (45). More effective anthropometric indicators such as chest circumference, waist-to-hip ratio, waist circumference measurement, hip circumference measurement, and waist-to-height ratio better gauge the health risk grade of obesity of individuals. Only one article (22) reported chest circumference as a clinical characteristic of its subjects. All included articles provided information about the subjects’ age distribution. Amongst the included articles, two articles (41, 43) only included elders as their subjects (mean age of 74.8), three articles (22, 40, 42) included both elders and young adults in their studies (in these three studies, all elders had comorbidities and all young subjects were healthy), and two articles (38, 39) included only subjects who were relatively young (10–60 years old). Six articles (22, 38, 40–43) provided health information about the subjects. While one of these six articles considered only healthy subjects (38), the other five (22, 40–43) included subjects with different combinations of comorbidities, which may have affected the morphology of the MCG signals collected compared to those of healthy patients.

It is important to consider the presence of different combinations of comorbidities when interpreting MCG signals, as comorbidities can introduce confounding factors that may affect the accuracy and reliability of the measurements and therefore the downstream HR predictions based on these measurements.

Only two studies (38, 39) reported accuracy/error rates of their HR estimations, one of which only included healthy subjects (38), while the health information of the subjects was not reported in the other (39). Therefore, for that study, we do not have information about whether any comorbidities were present to introduce irregularities to the signals and affect the accuracy of the HR estimations.

### Gyrocardiography setup and data

**Figure 3:**
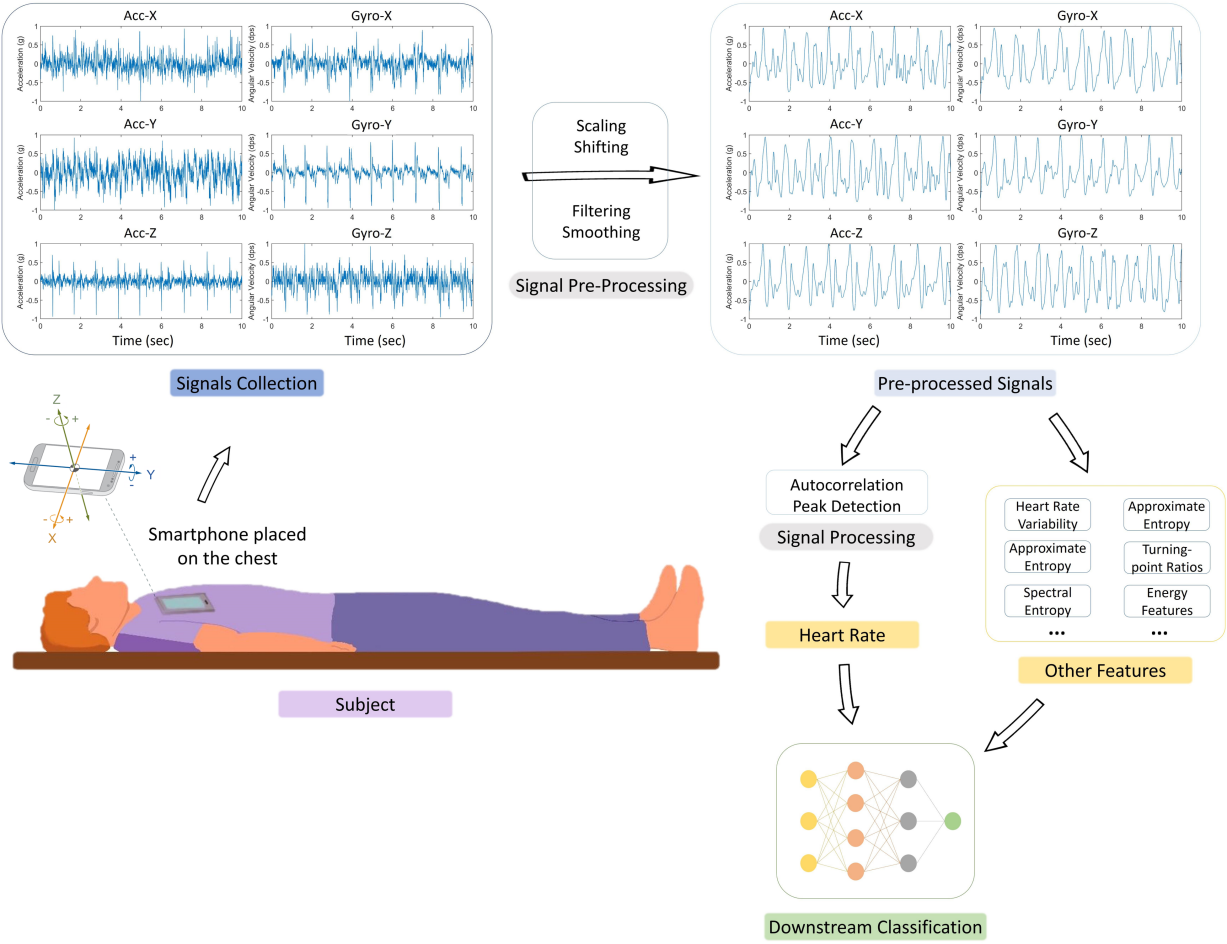
Experimental setup and signal processing pipeline in reviewed studies. This figure illustrates the generalized experimental setup and the signal processing steps employed in the seven articles analyzed in this review. Of these, two studies (38, 39) concluded their process at the Heart Rate (HR) detection stage post signal processing, while the remaining five (22, 40–43) proceeded to extract additional features for downstream classification, using the HR data alongside other features. The figure depicts the typical subject positioning (supine) during data collection using a smartphone, followed by the stages of raw signal pre-processing (scaling, shifting, filtering, and smoothing). The subsequent HR derivation predominantly involved techniques like short-term auto-correlation and peak detection. For performance evaluation, the same two studies (38, 39) focused on analyzing the accuracy and error rate of HR estimation compared to reference signals, while the others used the derived HR for further classification tasks related to conditions such as atrial fibrillation (AFib).

All the included studies adopted a similar experimental system setup. Figure 3 shows the visualization of the setup and the pipeline of the model, starting with data collection and data pre-processing, followed by HR derivation, and finally diverging either to perform downstream classification of heart conditions such as AFib or to directly assess the accuracy of the HR detection using evaluation metrics.

As shown in the smartphone model and the built-in sensors columns of Table 2, most of the studies (22, 38, 40–43) used both a tri-axial ACC and a tri-axial gyroscope with a sampling frequency of 200 Hz in a Sony Xperia smartphone. Only Mohamed et al. (39) conducted experiments using only a tri-axial gyroscope. In their study, Mohamed et al. (39) explored different smartphone models (Samsung Note II, Samsung S5, and Sony Z2) and found that Sony smartphone data, due to higher chip accuracy, provided the lowest error rate when used for HR estimation. One article (38) did not report the smartphone model used for data collection.

In terms of subject selection and measurement protocols, two articles (40, 42) provided details about the health criteria for participant inclusion and exclusion, and five studies (22, 40–43) declared that they had obtained approval for their research protocols from the ethical committees of the respective hospitals where patients’ data was collected.

All studies obtained their data through a dedicated data collection application. The data was all collected using smartphones placed on subjects’ chests in the supine position. Only Mohamed et al. (39) considered more than one measuring position, comparing the error rate of experiments in a supine position to those conducted holding the smartphone close to the chest in a sitting position, and concluded that the sitting position benefited the measurements more. None of the articles explored other vertical postures, such as standing or walking, which can lead to varied autonomic regulation of cardiovascular function and therefore different HR patterns to those of supine postures (46–48) due to an increase in the hydrostatic pressure of the thigh (49). All experiments, including the ones performed in the additional sitting position, involved placing the smartphone on or close to the chest. While placing the smartphone on the chest, whether in a sitting or supine position, is a reasonable position since the chest is the closest measuring site to the heart, this placement could also limit the usefulness of the application as it would be impractical for continuous ambulatory HR monitoring. The subjects were also instructed to remain still while lying in the supine position, which is very difficult in practical scenarios. In addition, it was required that the phone be put either on a bare chest or only light clothing, which makes convenient usage in cold weather conditions impossible.

For HR ground truth data, Huranen et al. (38) derived the reference HR from synchronized PPG signals collected by an externally synchronized sensor (sampling frequency =50 Hz), and Mohamad et al. (39) measured reference HR using an Omron HEM-432C blood pressure and pulse rate monitor. Jaakkola et al. (41), Tadi et al. (22), Iftikhar et al. (42) and Mehrang et al. (43) also collected synchronized ECG as their reference signal data, but these data were not utilized to obtain HR or assess HR prediction accuracy. Lahdenoja et al. (40) did not report any reference signal used in their studies.

### Signal processing

As illustrated in Figure 3, there are two main stages for HR detection using GCG: signal pre-processing and HR detection. Data pre-processing was applied for noise removal and data cleaning. The methods for these steps differ greatly in the different studies.

#### Pre-processing

Lahdenoja et al. (40) and Jaakola et al. (41) initiated their signal processing by individually pre-processing the six channels of the signals using a bandpass brick-wall Fast Fourier Transform (FFT) passband filter (1–45 Hz). This filtering step was crucial to remove noise and bias and to accentuate the cardiac motions in the signal.

Building upon a similar need for precise signal preparation, Mohamed et al. (39) took a different approach. They began by segmenting each gyroscope channel into fixed-size windows, each with a width “w”. Following this segmentation, they applied a local mean removal algorithm in each window, specifically targeting the elimination of the direct current component and enhancing the smoothness of the signal.

In a slightly varied technique, Hurnanen et al. (38) employed a second-order Butterworth Infinite Impulse Response bandpass filter (5–30 Hz) for each of the six channels of the MCG signal. Their aim was to eradicate high-frequency noise, signal offset, and trend. Additionally, they utilized a triangle-shaped finite impulse response filter (0.5s in length, 100 samples) to filter the absolute value of the signal, considering the roughly triangular envelopes of the heartbeat wavelets. The dynamic range of the signals was then balanced using the Successive Mean Quantization Transform (SMQT) algorithm, set at a quantization level of L=8, to facilitate peak detection by reducing the variance in peak amplitudes.

Echoing the methods of Lahdenoja et al. (40) and Jaakola et al. (41), Iftikhar et al. (42) adopted a similar preprocessing strategy. They also used a brick-wall FFT filter (1–40 Hz) for each measurement. To address the breathing component in the signal, they applied a mean filter (50 samples in length) to the original signal for estimation. This breathing component was then subtracted from the original signal, refining the focus on the cardiac-related aspects.

Tadi et al. (22) adopted a complex time series analysis method for their pre-processing. The signals from each of the six channels were first divided into segments of fixed lengths N. Instead of using frequency domain filters with pre-defined pass-band frequency limits, which can potentially cause the loss of vital information, Singular spectrum analysis (SSA) (50) was performed to decompose the signal into interpretable components such as periodic signals, noise, and baseline trend, as well as to further smooth the derived signals.

In addition to the SSA filter, Tadi et al. (22) performed envelope detection by filtering the signal firstly with a mean filter (4 samples in length), then by a triangular moving-average filter (8 samples in length), and finally by another triangular moving-average filter (51 samples in length). The triangular moving-average filters smooth out local fluctuations but keep long-term trends, therefore are able to capture stable cardiac waveforms for further assessment of cardiac rhythms.

Mehrang et al. (43) adopted the same filtering parameters (brick-wall FFT filter with cut-off frequencies of 1–40 Hz) as Lahdenoja et al. (40) and Jaakola et al. (41), and incorporated the breathing component removal step as proposed by Iftikhar et al. (42).

Two articles further elaborated on the artifact removal process for measurement inclusion of the acquired data. Lahdenoja et al. (40) used a sliding window Root Mean Square filter on the ACC Z (ACC-Z) axis to identify and eliminate artifacts because previous studies (51, 52) have demonstrated the high quality of the data obtained from the ACC-Z axis. The identification process of artifacts involves checking for the number of sample values in the filtered signal that exceed the median value of the ACC-Z signal. If this number is higher than a predefined threshold, the corresponding signal will be tagged artifact and subsequently discarded. Huranen et al. (38) eliminated the 8 measurements with low-quality PPG and used the 66 measurements with high-quality reference PPG for their experiments.

#### Heart rate estimation

The included articles performed HR estimation using each individual axis either from beat-to-beat duration detection using short-term Auto-Correlation (S-AC) (22, 40–43) or from local peak detection (38, 39).

For S-AC, overlapping sliding windows of size 2.5s (500 samples for sampling frequency = 200 Hz) were used for each 10s segment (2000 samples for sampling frequency = 200 Hz), resulting in 8 windows per segment. The output of each window represents the calculated lag of one heartbeat, which is used to calculate the corresponding HR. The median HR of the 8 estimated HR will be used as the final estimated HR for the segment.

Whereas for local peak detection, the respective methods detect the number of peaks in the processed signals as the number of heartbeats in the specified interval of the segment and estimate HR from these segments.

#### Post-processing

Two articles (38, 39) considered axis selection after predicting HR using each individual axis. While Hurnanen et al. (38) merely compared the performance of HR estimation using each axis and briefly described that the Gyro-Y axis has the lowest error rates, Mohamed et al. (39) adopted data post-processing which merges the axes using a Kalman filter and filters the merged predicted HR with an α-trimmed mean filter for more robust estimation.

After merging the three gyroscope axes and obtaining an HR estimate for each timestamp using a Kalman filter, an α-trimmed mean filter (30 samples in length) is used for a more robust HR estimation, where α defines the extent to which the filter behaves like a median filter.

#### Performance evaluation metrics

Only two articles (38, 39) conducted a performance evaluation for HR estimation. The other five articles (22, 40–43) focused on downstream classification using HR as a feature. Mohamed et al. (39) used HR measured from a pulse rate monitor as the reference to evaluate the accuracy of their HR estimation, while Hurnanen et al. (38) used a PPG signal as the reference. The evaluation metrics used were also inconsistent among the studies. While Mohamed et al. (39) calculated median average error (beats per minute), Hurnanen et al. (38) calculated the numbers and percentages of missed and false beats estimated.

Performance evaluation metrics are imperative for ensuring that HR estimation methods are evaluated based on their accuracy and precision. Accuracy and precision are critical when making decisions that affect people’s health and safety. It is also important to have consistent metrics for evaluating and comparing the performance of different HR estimation methods objectively. Objectivity improves the reliability of methods and allows for the identification of the most effective and efficient methods.

## Discussion: interpretation of results, challenges, and future directions

An important aspect of our review that warrants discussion is the observation that a significant portion of the literature, specifically six out of the seven articles reviewed, were authored by a consistent group of researchers. We recognize that this concentration of research output from a single group might raise questions about the representativity of our review’s findings and the generalizability of the conclusions drawn. To address this, we have conducted a detailed analysis of the methodologies and datasets used in these studies to uncover any underlying dissimilarities or unique approaches that may exist. This examination is crucial to understanding the breadth and depth of the research conducted by this group and to ascertain whether their findings and methodologies could be considered as representative of the field’s current state. The results of this inquiry, focusing on the diversity in datasets and methodological approaches, are discussed comprehensively in the dataset demographics section and the methodology discussion in our manuscript. By doing so, we aim to present a balanced and objective view of the current research landscape in gyrocardiography, acknowledging the contributions of this prominent group while also critically evaluating the impact of their dominance in the field on the overall conclusions of our review.

Our literature search showed that HR estimation using smartphone GCG data is feasible, with many studies taking this further to use HR estimation for downstream classification applications. However, it should be noted that while our literature search began in 2012, the significant body of research specifically focusing on gyrocardiography for HR estimation has emerged more recently, from around 2017, indicating the novelty of this application in the field.

First, inconsistency exists in the findings as to whether using gyroscope data alone or combining it with ACC data produces higher accuracy in HR detection. Lahdenoja et al. (40) and Hurnanen et al. (38) claim that using both GCG and SCG leads to better results, while Mohamed et al. (39) report that using GCG alone yields better results than using both. These inconsistencies may be due to the different data processing methods used across the studies. In addition, different data quality and individual physiology may play roles in HR estimation accuracy. For example, some methods might be more sensitive to higher HR and therefore be better able to estimate HR in certain individuals with overall higher HR. Most importantly, while Lahdenoja et al. (40) and Hurnanen et al. (38) hypothesized that using all six axes (tri-axial ACC and tri-axial gyroscope) should yield more accurate HR estimation, they did not conduct performance evaluations on HR estimation, and the subject pool used by Mohamed et al. (39) to support their claim was relatively small. Therefore, additional research is needed to fully understand the potential benefits and limitations of using gyroscopes with and without ACCs for HR detection.

A major challenge in this field is data collection. Factors such as subject selection, dataset size, data quality, ethical considerations, and protocol standardization have to be taken into account during the data collection process for holistic research. The accuracy of HR estimation methods may vary across different populations with different ages, genders, ethnicities, medications, obesity levels, and comorbidities due to differences in physiology and lifestyle. Therefore, it is important to ensure that the methods used for HR estimation are validated in diverse populations to guarantee applicability and accuracy for all individuals. The diversity of the subject population mandates that the number of subjects included in the studies should not be too small. For example, while Mohamed et al. (39) claimed to have achieved better than state-of-the-art accuracy, the size of their subject pool was less than 20. Their methods, therefore, need to be validated on a larger dataset to prove their robustness. To ensure the reliability of the results of a study, the data collected from smartphone sensors should be of high quality and free from noise and artifacts to facilitate signal processing tasks. This requires careful selection of the smartphone model used, which would require experiments on more smartphone models.

Furthermore, while our study focuses on the utilization of smartphones for medical data collection, it is important to recognize the broader context. Traditional methods of medical data collection often involve more specialized equipment and trained personnel, making them more expensive and time-consuming compared to the smartphone-based methods explored here. This comparison underscores the potential of smartphones as a more accessible and less resource-intensive alternative for HR data collection, which could be particularly beneficial in resource-limited settings or for widespread population health monitoring.

Fortunately, efforts have been made toward facilitating medical data collection and sharing for research purposes. Hospitals are collaborating with institutions to provide access to medical datasets and facilitate research in various domains. As seen in Figure 2, there has been a significant increase in dataset size (from 39 subjects to ≥ 300 subjects) in this area of research. Additionally, with the increasing prevalence of smartphones, there may be more opportunities to collect GCG signals in a non-invasive and convenient manner. This could potentially lead to larger and more diverse datasets for HR estimation that will enable the development of more robust and reliable models for HR estimation.

All included articles, except Mohamed et al. (39), used GCG signals collected only when the user was in a supine position, with the phone placed flat on the user’s chest. Only collecting data from a supine position may limit the use of these collection methods as ambulatory monitors in real-life scenarios where subjects are less likely to remain stationary. Different postures and physical activity patterns should be considered and studied, as they can lead to differences in the measured GCG signals due to changes in pressure on the chest. Future methods may need to combine activity recognition with HR estimation to take the level of physical activity into account.

A gold standard for performance evaluation is notably lacking in the included articles. While in medicine, ECG is the gold standard for HR monitoring, Jaakkola et al. (41), Tadi et al. (22), and Mehrang et al. (43) used ECG as a reference signal, Hurnanen et al. (38) used PPG as a reference signal, and Mohamed et al. (39) used HR readings from a blood pressure monitor as a reference signal. A lack of standardization in terms of data collection, analysis methods, and performance evaluation makes it difficult to compare results across studies. Establishing standard protocols for data collection and analysis is therefore essential for advancing this field.

We provide a list of recommendations for addressing the above challenges, which will require a multidisciplinary approach involving expertise in signal processing, physiology, pathophysiology, medicine, and novel method development.

• Standardize the gold standard (e.g., ECG) for a fair comparison against a reliable reference.
• Standardize the metric for convenient performance evaluation.
• Perform methods evaluation using the standardized reference signals and metrics.
• Compare the effect of using six-axial data vs using tri-axial gyroscope data only.
• Collect data at different positions when the subjects are in different postures and study the effect of postures and data collection sites on HR detection accuracy.
• Incorporate physical pattern recognition into the algorithm.
• Include subjects with a more diverse background in terms of gender, ethnicity, and comorbidity.

Much more research and development is needed before the use of GCG signals can be widely adopted in healthcare settings.

## Conclusions

In this scoping review, we conducted a literature search on HR detection using smartphone gyroscope data. We have described the data processing methods used in each article, presented challenges, and potential approaches to these challenges, suggested future research directions, and discussed the standardization required in this field. Despite the potential usefulness of using gyroscopes for HR detection, there are relatively few literature articles dedicated to researching this purpose. This could be largely attributable to a lack of awareness about the potential to use gyroscopes for HR detection, as ACCs have previously been the most popular tool for this purpose. It is hoped that this review will increase researchers’ awareness about the potential of using smartphone GCG for HR estimation.
